# Impact of Three-Dimensional Assessment of Maternal Left Ventricular Systolic Function on Pregnancy Outcomes

**DOI:** 10.31083/RCM27639

**Published:** 2025-04-17

**Authors:** Aleksandra Ilić, Snežana Tadić, Maja Stefanović, Djordje Ilić, Milovan Petrović, Aleksandra Milovančev, Marija Bjelobrk, Tatjana Miljković, Dragana Dabović, Snežana Stojšić, Muamer Bačevac, Anastazija Stojšić-Milosavljević

**Affiliations:** ^1^Faculty of Medicine, University of Novi Sad, 21000 Novi Sad, Serbia; ^2^Institute of Cardiovascular Diseases of Vojvodina, 21208 Sremska Kamenica, Serbia; ^3^Department of Obstetrics and Gynecology, Clinical Center of Vojvodina, 21000 Novi Sad, Serbia; ^4^Department of Internal Medicine, Opšta bolnica Novi Pazar, 36300 Novi Pazar, Serbia

**Keywords:** 3D echocardiography, global area strain, hypertension in pregnancy, pregnancy outcome

## Abstract

**Background:**

Hypertensive disorders in pregnancy (HDP) are associated with adverse pregnancy outcomes. Three-dimensional (3D) echocardiography provides greater accuracy for assessing cardiac geometry and function during pregnancy. The aim was to assess the impact of the 3D left ventricle (LV) systolic function in HDP on pregnancy outcomes.

**Methods:**

The prospective cohort study included primiparous with singleton pregnancies, without previous comorbidities who underwent medical history assessment, laboratory tests, ambulatory blood pressure monitoring (ABPM), and transthoracic echocardiography at baseline and six weeks after delivery. Participants were divided into a HDP group and a control group. Pregnancy outcomes (intrauterine growth restriction (IUGR), preterm delivery, and birth weight) were recorded and analyzed.

**Results:**

The study involved 174 HDPs and 64 controls, with a median gestational age of 34 weeks (31; 36). Compared to controls HDP exhibited significantly impaired values in both two-dimensional (2D) and 3D parameters for the systolic and diastolic function of the LV. They had higher LV mass index values and lower absolute values for 2D global longitudinal strain and 3D LV strain in all directions (*p* < 0.001). Multivariable regression analysis revealed that body mass index (BMI) with odds ratio (OR) of 0.751 (95% confidence interval (CI): 0.666–0.847, *p* < 0.001) and 3D LV global area strain (GAS) with OR of 0.234 (95% CI: 0.155–0.352, *p* < 0.001) were the strongest predictors of IUGR, while BMI with OR of 0.832 (95% CI: 0.758–0.914), nighttime systolic blood pressure (SBP) with OR of 1.055 (95% CI: 1.032–1.079, *p* < 0.01) and 3D LV ejection fraction (EF) with OR of 0.780 (95% CI: 0.687–0.885) were the strongest predictors of preterm delivery. The receiver operating characteristic (ROC) curve showed that the model with BMI and 3D LV GAS can be a good predictor for IUGR with an area under the curve (AUC) 0.951 (0.925–0.976) with 89.5% sensitivity and 86.4% specificity, *p* < 0.001, while the model with BMI, nighttime SBP and 3D LV EF is a predictor for preterm delivery with AUC of 0.835 (0.776–0.893) with 79.1% sensitivity and 73.7% specificity, *p* < 0.001. Person correlation showed a significant positive correlation between birth weight and 3D GAS, r = 0.485; *p* < 0.001.

**Conclusions:**

LV GAS is significantly associated with IUGR and birth weight, while 3D LV EF strongly predicts preterm delivery.

## 1. Introduction 

Hypertensive disorders in pregnancy (HDP) are associated with adverse pregnancy outcomes, both for the mother and the newborn. Also, HDP is a risk factor for ischemic heart disease, hypertension, and stroke later in life [1].

During pregnancy, the mother’s heart adapts to the increased volume, usually changing its geometry toward eccentric hypertrophy or concentric remodeling leading to changes in its function [2]. As echocardiographic parameters for assessing geometry and systolic function are volume-dependent they may not entirely reflect left ventricular geometry in pregnant women. The impacts of these changes are more evident in pregnancies affected by gestational hypertension and preeclampsia (PE). Consequently, in addition to physiological changes, these alterations in maternal cardiac geometry in women with HDP are linked to more pronounced adverse remodeling. This remodeling can further influence cardiac function and contribute to the overall risk of adverse pregnancy outcomes, highlighting the importance of thorough cardiac assessment during pregnancy [3]. Three-dimensional (3D) echocardiography has proved to be highly precise for evaluating cardiac chamber geometry [4]. During pregnancy, slight systolic function decline is observed, as indicated by both ejection fraction (EF) and left ventricular global longitudinal strain (LV GLS) [5]. Similar changes were also evident in the 3D assessment of maternal cardiac morphology and left ventricular myocardial deformation, which showed a better correlation with magnetic resonance imaging (MRI) than two-dimensional (2D) assessment [6]. On the other hand, studies have shown that 3D LV GLS is a better predictor of adverse events compared to EF and 2D LV GLS in patients who have experienced a myocardial infarction [7], asymptomatic diabetic patients compared to control [8], as well as in cardio-oncology [9].

There is limited data assessing a mother’s 3D left ventricular systolic function during pregnancy. While 3D echocardiography (3DE) has been used in other clinical scenarios, its application in HDP is still underexplored. 3DE has shown significant advantages in accuracy, reproducibility, and versatility, often serving as a stronger prognostic predictor than two-dimensional echocardiography (2DE). The assessment of chamber volumes using 3DE has been more precise and accurate. Parameters such as ejection fraction and left ventricular hypertrophy are recognized as significant predictors of cardiovascular outcomes. However, despite their importance in cardiovascular pathology, the use of 3DE to assess cardiac geometry during pregnancy has not been extensively utilized or studied.

The aim was to assess the impact of the 3D left ventricle systolic function in women with HDP on pregnancy outcomes.

## 2. Materials and Methods

This prospective cohort study enrolled primiparous women with singleton pregnancies who had no previous comorbidities or risk factors. Pregnant women with any pregnancy-related comorbidities, such as gestational diabetes or thyroid disorders, were excluded from the study.

Conducted from December 2021 to April 2024 at a tertiary care center, the study involved a comprehensive assessment of each participant’s medical history, physical examination, laboratory tests, transthoracic echocardiogram, and 24-hour ambulatory blood pressure monitoring (ABPM). This was done both at baseline (screening) between the 23rd and 41st gestational weeks, with a median of 34.0 (ranging from 31.0 to 36.0 weeks) for the entire cohort, and again six weeks after delivery. The goal was to exclude any cases of chronic (pre-existing) hypertension. Six weeks postpartum, all participants were normotensive; those with still-elevated blood pressure values were excluded from the study.

A total of 321 pregnant women were screened. After excluding 18 subjects due to insufficient image quality for analysis, 22 for incorrectly recorded blood pressure by ABPM, and 43 who were lost to follow-up (as they did not attend check-ups), 174 women with HDP and 64 normotensive healthy pregnant controls were analyzed.

In this study, “HDP” refers to gestational hypertension and PE, defined according to the current guidelines of the European Society of Cardiology [1]. The research received approval from the Institution’s Ethics Committee (number 1189-1/5), and all participants provided informed consent to take part in the study.

### 2.1 24 h ABPM

ABPM was performed during screening, six weeks after delivery, and also throughout pregnancy to monitor the blood pressure (BP) values of pregnant women undergoing treatment for high blood pressure. ABPM was obtained using the Meditech Cardio Tens device (Meditech Ltd., Budapest, Hungary).

Blood pressure measurements were recorded every 20 minutes from 6 am to 10 pm, and every 30 min from 10 pm to 10 am the next day. The average values ​for daytime systolic and diastolic BP, as well as nighttime systolic and diastolic BP and heart rate (HR), were used for analysis.

### 2.2 Echocardiography

#### 2.2.1 Two-Dimensional Echocardiography

All patients underwent resting transthoracic echocardiography in the left lateral position at screening and again 6 weeks after delivery. Images were captured using a GE Vivid E95 ultrasound machine (GE, SN 0BB459D4 version 204, Milwaukee, WI, USA) equipped with a M5Sc-D transducer 1.5–4.5 MHz (2D) with simultaneous electrocardiogram monitoring. The investigator, who was blinded to the clinical characteristics of participants, analyzed three cardiac cycles in sinus rhythm using uncompressed data that were stored in cine-loop format on EchoPAC PC v SN 0BB459D4 version 204 GE, Milwaukee, WI, USA.

The evaluation included 2D volumes, mass, both systolic and diastolic function of the left ventricle, as well as 2D LV GLS in accordance to current recommendations [10, 11]. 2D LV GLS was obtained in the apical 2-, 3-, and 4-chamber views and calculated by averaging the peak systolic strain values in the myocardial segments. Mass and cardiac output were indexed by body surface area (BSA).

#### 2.2.2 Three-Dimensional Echocardiography

For 3D analysis with a 4Vc-D 1.5–5 MHz (3D) transducer full-volume images were captured from an apical view with 6 second single breath holds to avoid stitching. All the optimal data sets were recorded in a raw-data format and exported to a workstation where 3D full-volume data sets were analyzed using the 4D Auto Left Ventricular Quantification (LVQ) package on the Echopack workstation. Acquisitions were made with volume rates of 20–40 volumes per second. The endocardial border and the epicardial surface were acquired automatically. All captured images were stored digitally for later analysis. If tracking was considered suboptimal, the endocardial border was manually corrected. Then left ventricular (LV) volumes, ejection fraction, LV mass, and cardiac output were calculated automatically.

3D speckle tracking echocardiography of the left ventricle was estimated from a 12-slice, multi-beat, single 3D full-volume data set as 3D LV GLS, global circumferential strain (3D LV GCS), global radial strain (3D LV GRS), and global area strain (3D LV GAS) by using the 4D Auto LVQ function in the software [4, 6, 12, 13]. Patients with suboptimal images and with three or more rejected segments were excluded from the study.

### 2.3 Endpoints

Participants were followed and pregnancy endpoints were recorded: intrauterine growth retardation (IUGR), preterm delivery, and birth weight. IUGR was defined as a newborn’s weight below the 10th percentile for its gestational age [14]. Delivery before the 37th gestational week (GW) was considered as preterm delivery [15].

### 2.4 Statistical Analysis

Categorical variables are presented as absolute numbers and percentages. The Kolmogorov-Smirnov test was used to test the normal distribution. Continuous variables are presented as the means and standard deviations or median with interquartile ranges (25th and 75th percentile). Differences between groups were tested via Student’s paired *t*-test or Mann-Whitney test, ANOVA, Wilcoxon, and the chi-square test as appropriate. Linear Pearson’s correlation test was used for the assessment of the influence of the studied variables on birth weight. Binary logistic regression was used to determine independent predictors of primary endpoints, and these were expressed as estimated odds ratios (ORs) with their corresponding 95% confidence intervals (CIs). Variables found to be statistically significant by an univariable analysis were used for the multivariate model building, and *p*-values lower than 0.05 were considered statistically significant. The fitting effect of multivariate binary logistic regression analysis was evaluated by the receiver operating curve (ROC). The statistical software Statistica (Statistica 13.5, The Ultimate Academic Bundle, StatSoft Europe GmbH, Hamburg, Germany; university license for the University of Novi Sad) was used for all analyses.

## 3. Results

### 3.1 Demographic Characteristics, Findings of Ambulatory Blood Pressure Monitoring, and Echocardiographic Examination at Screening

A total of 238 pregnant women were included in the analysis, out of which 174 had HDP, among these 129 met the criteria for gestational hypertension (GH) and 45 for PE, while 64 were normotensive healthy pregnant controls. The median age of all participants was 31 years (26; 35). Women with HDP were significantly older than controls, *p* = 0.018. At baseline, the median gestational week was 34 (31; 36).

The baseline screening characteristics of all participants are shown in Table 1. Women with HDP exhibited a statistically significant reduction in both 2D and 3D parameters of systolic and diastolic function. They also had significantly higher values for the left ventricular mass index (*p* < 0.001), without a significant difference in cardiac output (2D: *p* = 0.302; 3D: *p* = 0.843). Also, the absolute values ​​of 2D LV GLS were significantly lower in the HDP group, as were the values ​​of 3D strain in all directions compared to the control (*p* < 0.001).

**Table 1.** **Baseline patients characteristics**.

Parameter	All (N = 238)	HDP (N = 174)	Controls (N = 64)	*p*
	Median (IQR)	Median (IQR)	Median (IQR)	
GW	34 (31; 36)	36 (34; 38)	33 (31; 35)	<0.001
Age (years)	31 (26; 35)	32 (27; 35)	29 (25; 33.5)	0.018
Height (cm)	167 (164; 170)	168 (164; 170)	165 (164; 171)	0.236
Weight (kg)	79.5 (73; 90)	82.5 (75; 92)	75 (71; 78)	<0.001
BMI (kg/m^2^)	29.05 (27.16; 32.62)	30.3 (27.68; 33.39)	27.39 (26.08; 27.72)	<0.001
SBPav daytime (mmHg)	140 (124; 146)	145 (139; 150)	119 (115; 122)	<0.001
SBPav nighttime (mmHg)	128 (108; 139)	134 (126; 142)	105 (98; 107)	<0.001
DBPav daytime (mmHg)	88 (77; 93)	92 (87; 95)	70 (68; 75)	<0.001
DBPav nighttime (mmHg)	74 (62; 89.25)	82 (73; 92)	58 (55; –61.5)	<0.001
Average HR (beat per minute)	88 (82; 98)	88 (82; 98)	88 (82; 99)	0.740
E (m/sec)	0.80 (0.70; 0.90)	0.80 (0.70; 0.90)	0.90 (0.80; 1.04)	<0.001
e’s (m/sec)	0.09 (0.08; 0.11)	0.09 (0.08; 0.10)	0.12 (0.10; 0.13)	<0.001
e’l (m/sec)	0.11 (0.09; 0.14)	0.10 (0.09; 0.12)	0.15 (0.13; 0.17)	<0.001
E/e’av	8.1 (6.92; 8.75)	8.42 (7.37; 8.89)	6.95 (6.21; 7.96)	<0.001
2D LV EDV (mL)	96 (85; 109)	97 (85; 110)	95 (85; 108.5)	0.452
2D LV ESV (mL)	35.5 (31.38; 40)	36 (32; 40)	33.5 (28; 40)	0.034
2D LV EF (%)	63.07 (62; 64.84)	62.90 (61; 64.75)	63.92 (62.44; 64.89)	<0.001
2D CO index (L/min/m^2^)	2.97 (2.66; 3.40)	2.96 (2.62; 3.46)	3.01 (2.80; 3.31)	0.302
2D mass index (g/m^2^)	84.59 (75.42; 97.13)	91:3 (83.24; 98.92)	74.84 (72.16; 77.62)	<0.001
3D LV EDV (mL)	99 (89; 116)	99.5 (91; 117)	98 (88; 110)	0.059
3D LV ESV (mL)	38 (33; 43)	40 (35; 44)	35 (29; 40.5)	<0.001
3D LV EF (%)	62.71 (61; 64.62)	61.99 (60.32; 63.72)	64.09 (62.72; 64.82)	<0.001
3D CO index (L/min/m^2^)	3.15 (2.71; 3.55)	3.18 (2.67; 3.64)	3.15 (2.9; 3.35)	0.843
3D mass index (g/m^2^)	85.98 (76.11; 97.96)	91.76 (83.17; 101.02)	75.34 (73.76; 78.72)	<0.001
2D LV GLS	–19 (–18.9; –20.6)	–19.15 (–18; –22.0)	–20.6 (–20.2; –21.1)	<0.001
3D LV GLS	–17.3 (–15.9; –19.2)	–16.5 (–15.9; –18)	–20.55 (–19.4; –21)	<0.001
3D LV GCS	–19.1 (–18.55; –20.4)	–16.8 (–15.9; –17.4)	–19.8 (–18.2; –21.1)	<0.001
3D LV GRS	48.55 (45.1; 51.9)	46.2 (44.9; 49.2)	53 (51.9; 54.05)	<0.001
3D LV GAS	–30.7 (–27.78; –32.6)	–26.8 (–26; –37.2)	–32.5 (–31.8; –32.8)	<0.001

HDP, hypertensive disorders in pregnancy; GW, gestational week; BMI, body mass index; IQR, interquartile range; SBPav, systolic blood pressure average value; DBPav, diastolic blood pressure average value; HR, heart rate; E, transmitral early peak velocity; e’s, early diastolic mitral annulus septal velocity; e’l, early diastolic mitral annulus lateral velocity; E/e’av, left ventricle filling pressure; 2D, two-dimensional; 3D, three-dimensional; LV, left ventricle; EDV, end-diastolic volume; ESV, end-systolic volume; EF, ejection fraction; CO, cardiac output; 2D LV GLS, two-dimensional left ventricular global longitudinal strain; 3D LV GLS, three-dimensional left ventricular global longitudinal strain; 3D LV GCS, three-dimensional left ventricular global circumferential strain; 3D LV GRS, three-dimensional left ventricular global radial strain; 3D LV GAS, three-dimensional left ventricular global area strain; *p*-value, HDP vs Controls. Interquartile range (25-percentile; 75th percentile).

### 3.2 Demographic Characteristics, Findings of Ambulatory Blood Pressure Monitoring, Echocardiographic Examination Six Weeks After Delivery, and Pregnancy Outcomes

Pregnancy outcomes and participants’ characteristics after delivery are presented in Table 2. Women who were hypertensive during pregnancy, although normotensive after delivery, when compared to controls still had statistically significantly higher BP values (*p* < 0.001). A significant difference was also observed after delivery in the filling pressure of the left ventricle E/e’av (*p* < 0.001). Likewise, women with HDP had significantly higher LV mass values ​​than controls (*p* < 0.001). Regarding the LV systolic function, the difference in EF between the two groups observed before delivery was no longer significant after delivery, with 2D imaging showing a *p*-value of 0.163 and 3D imaging showing a *p*-value of 0.891. However, 2D and 3D strain values remained worse in women with HDP (*p* < 0.001). Pregnant women with hypertension were more likely to give birth prematurely compared to those without hypertension (*p* < 0.001). Their infants also had lower birth weights and experienced intrauterine growth restriction more frequently (*p* < 0.001).

**Table 2.** **Patient characteristics, ambulatory blood pressure monitoring parameters, echocardiography after delivery, and pregnancy outcomes**.

	All (N = 238)	HDP (N = 174)	Controls (N = 64)	*p*
	Median (IQR)	Median (IQR)	Median (IQR)	
	N (%)	N (%)	N (%)	
Weight (kg)	69 (65; 82)	71 (67; 85)	65 (64; 69)	<0.001
BMI (kg/m^2^)	24.8 (23.42; 29.02)	25.91 (23.88; 30.59)	23.8 (22.76; 24.77)	<0.001
SBPav daytime (mmHg)	124 (118; 126)	125 (123; 127)	116.5 (115; 119)	<0.001
SBPav nighttime (mmHg)	108.5 (103.75; 114)	112 (107; 116)	103 (100; 105)	<0.001
DBPav daytime (mmHg)	76 (73; 77)	76 (75; 78)	71 (65; 72.5)	<0.001
DBPav nighttime (mmHg)	64 (59; 66)	65 (63; 66)	58 (55; 59)	<0.001
Average HR (beat per minute)	81 (71; 87)	72 (76; 89)	74 (70; 85.5)	<0.001
E (m/sec)	0.9 (0.80; 1)	0.9 (0.8; 1.03)	0.9 (0.8; 1)	0.950
e’s (m/sec)	0.12 (0.11; 0.14)	0.12 (0.11; 0.14)	0.12 (0.11; 0.14)	0.547
e’l (m/sec)	0.15 (0.13; 0.17)	0.15 (0.13; 0.16)	0.16 (0.14; 0.18)	<0.001
E/e’av	7.10 (6.21; 7.64)	7.20 (6.40; 7.83)	6.56 (6.03; 7.3)	<0.001
2D LV EDV (mL)	91 (84.25; 99)	91 (85; 99)	91 (82; 98)	0.403
2D LV ESV (mL)	32 (31; 35.25)	32 (31; 36)	31 (28.5; 35)	0.050
2D LV EF (%)	64.29 (63; 65.19)	64.29 (63; 65.05)	64.29 (63.38; 65.45)	0.163
2D CO index (L/min/m^2^)	2.91 (2.67; 3.14)	2.97 (2.68; 3.17)	2.81 (2.63; 2.93)	<0.001
2D mass index (g/m^2^)	71.85 (68.52; 76.59)	72.79 (68.70; 77.64)	70.4 (68.49; 74.16)	<0.001
3D LV EDV (mL)	94 (85; 99)	91 (85; 99)	94 (85; 99)	0.957
3D LV ESV (mL)	31 (29; 35)	31 (29; 36)	33 (28.5; 34)	0.963
3D LV EF (%)	65.56 (64.21; 67.03)	65.56 (64.20; 67.03)	65.66 (64.36; 66.50)	0.891
3D CO index (L/min/m^2^)	2.95 (2.78; 3.22)	3.05 (2.81; 3.35)	2.89 (2.68; 2.92)	<0.001
3D mass index (g/m^2^)	73.13 (68.61; 77.26)	74.1 (68.92; 79.17)	71.49 (68.54; 74.51)	<0.001
2D LV GLS	–20.2 (–19.90; –21.23)	–20 (–19.8; –20.6)	–21.6 (–20.8; 22)	<0.001
3D LV GLS	–22.5 (–19.78; –23.80)	–22.05 (–19.6; –23.8)	–22.9 (–21.5; –23.9)	<0.001
3D LV GCS	–19.8 (–19.10; –22.03)	–19.4 (–19; –20.8)	–21.6 (–20.35; –22.7)	<0.001
3D LV GRS	54.3 (53.78; 55.90)	54.1 (53.4; 55.3)	55.8 (54.30; 56.1)	<0.001
3D LV GAS	–35.4 (–34.50; –36.80)	–27.1 (–25.5; –28.6)	–36.1 (–34.75; –36.9)	<0.001
Birth weight (g)	2900 (2380; 3450)	2770 (2250; 3210)	3330 (2800; 3710)	<0.001
IUGR	76 (31.9)	74 (42.5)	2 (3.1)	<0.001
Preterm delivery	67 (28.2)	58 (33.3)	9 (14.1)	<0.001

HDP, hypertensive disorders in pregnancy; BMI, body mass index; SBPav, systolic blood pressure average value; IQR, interquartile range; DBPav, diastolic blood pressure average value; HR, heart rate; E, transmitral early peak velocity; e’s, early diastolic mitral annulus septal velocity; e’l, early diastolic mitral annulus lateral velocity; E/e’av, left ventricle filling pressure; 2D, two-dimensional; 3D, three-dimensional; LV, left ventricle; EDV, end-diastolic volume; ESV, end-systolic volume; EF, ejection fraction; CO, cardiac output; 2D LV GLS, two-dimensional left ventricular global longitudinal strain; 3D LV GLS, three-dimensional left ventricular global longitudinal strain; 3D LV GCS, three-dimensional left ventricular global circumferential strain; 3D LV GRS, three-dimensional left ventricular global radial strain; 3D LV GAS, three-dimensional left ventricular global area strain; IUGR, intrauterine growth restriction; *p*-value, HDP vs Controls. Interquartile range (25-percentile; 75th percentile).

### 3.3 Outcomes

#### 3.3.1 Birth Weight

**Fig. 1.**
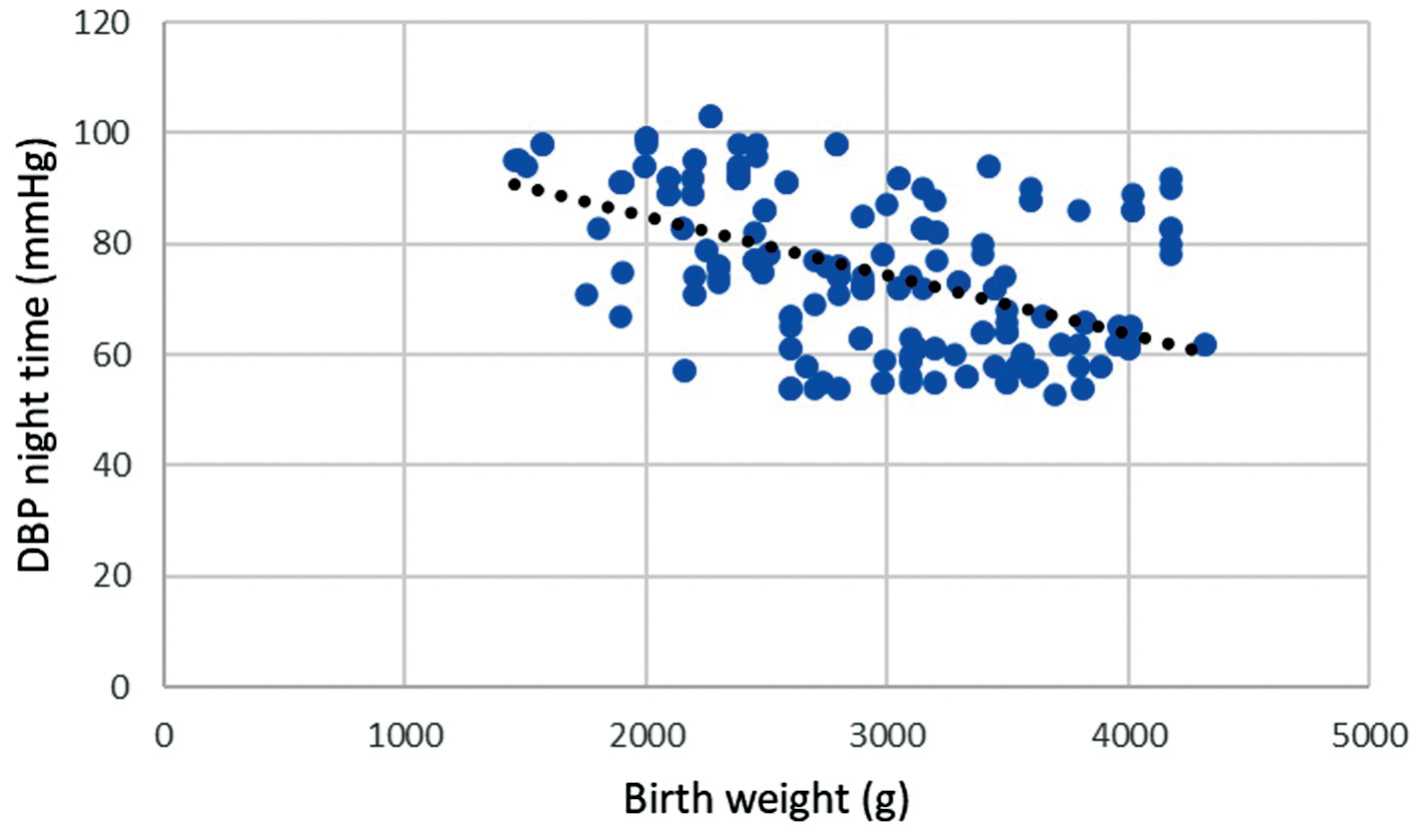
**Correlation of birth weight and night-time diastolic blood pressure during pregnancy**. Legend: Scatter plot showing significant negative strong correlation between birth weight and night-time diastolic blood pressure (DBP), r = –0.502; *p* < 0.001.

**Fig. 2.**
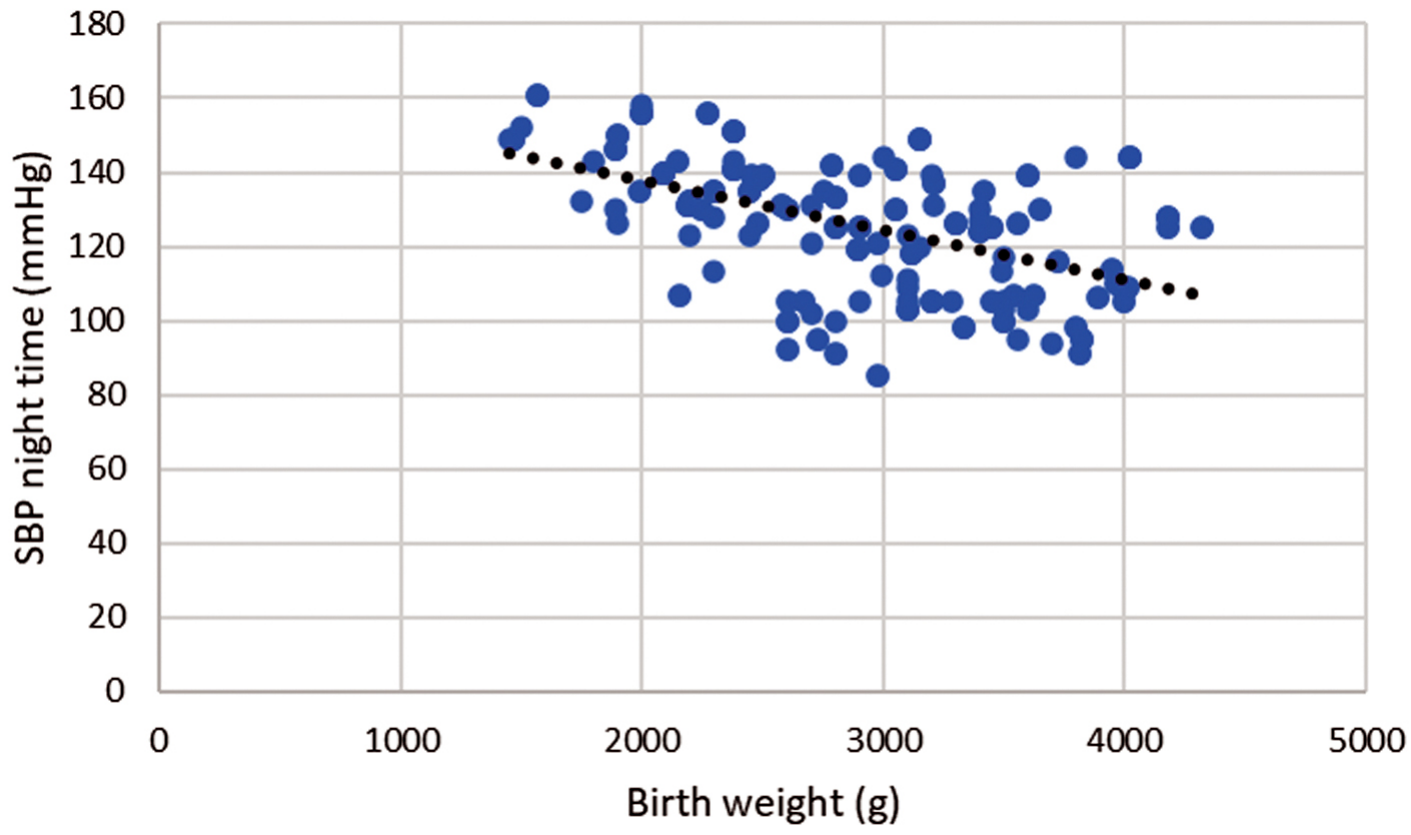
**Correlation of birth weight and night-time systolic blood pressure during pregnancy**. Legend: Scatter plot showing significant negative strong correlation between birth weight and night-time systolic blood pressure (SBP), r = –0.506; *p* < 0.001.

Based on the Pearson correlation coefficient there is a statistically significant negative moderate association between birth weight and night-time diastolic BP, r = –0.502; *p* < 0.001. This means that as night-time diastolic BP increases birth weight decreases, and approximately 25.2% of the variance in birth weight can be explained by changes in night-time diastolic BP. Pearson correlation coefficient shows a negative linear correlation for night-time systolic BP, r = –0.506; *p* < 0.001, as night-time systolic BP increases birth weight decreases, thus, about 25.6% of the variance in birth weight is explained by changes in night-time systolic BP (Figs. 1,2).

**Fig. 3.**
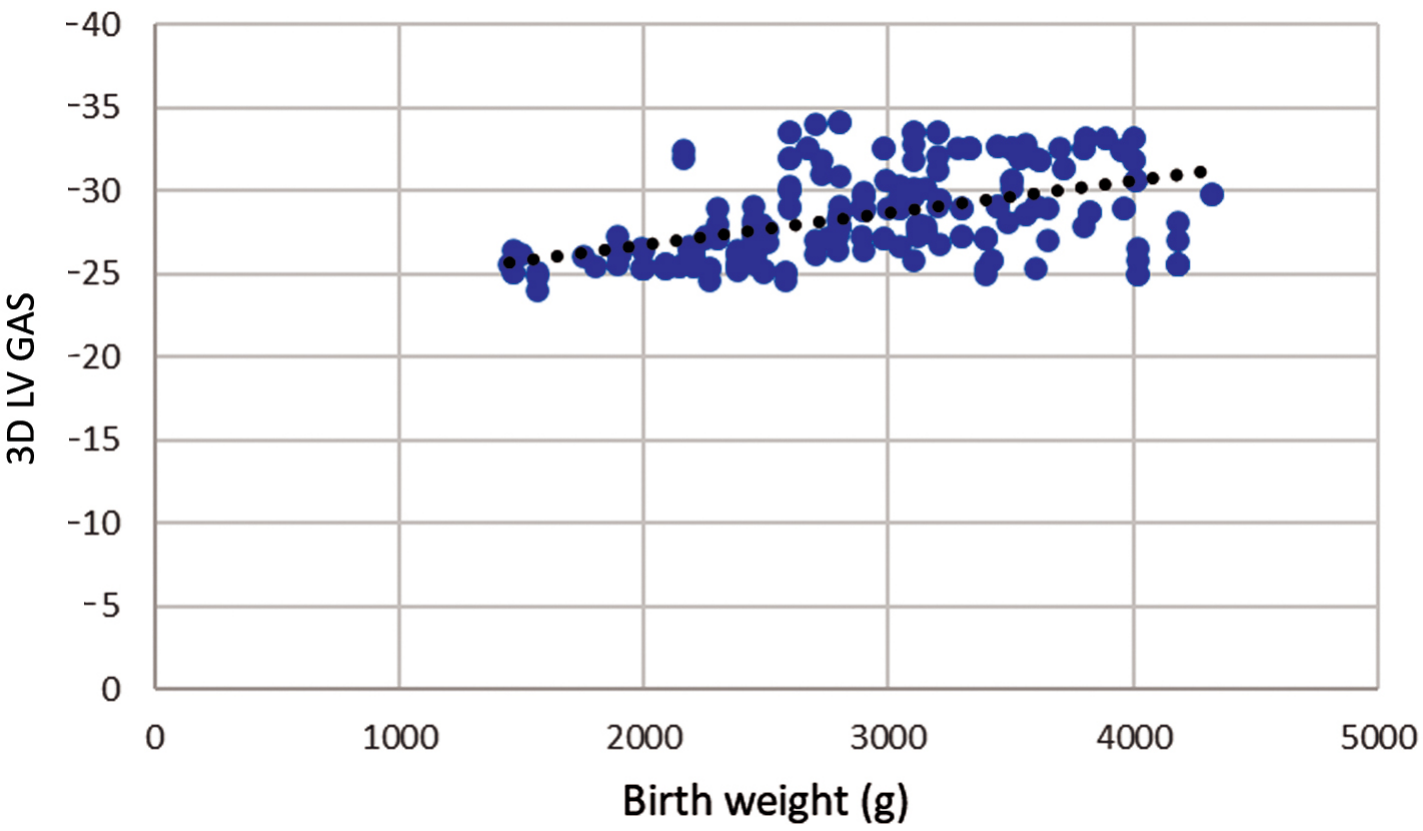
**Correlation of birth weight and three-dimensional left ventricular global area strain during pregnancy**. Legend: Scatter plot showing significant positive strong correlation between birth weight and three-dimensional left ventricular global area strain (3D LV GAS), r = 0.485; *p* < 0.001.

There is also a statistically significant moderate positive correlation between birth weight and absolute values ​​of 3D GAS r = 0.485; *p* < 0.001 (Fig. 3), as 3D LV GAS decreases birth weight decreases.

#### 3.3.2 IUGR

The impact of HDP group parameters on IUGR was analyzed.

Univariate regression analysis showed that fetal growth retardation is influenced by an increase in body mass index, age, blood pressure, left ventricular mass index, and left ventricular systolic function, assessed by both two- and three-dimensional echocardiography. Multivariable regression analysis revealed that the strongest predictors for the occurrence of IUGR are body mass index (BMI) and 3D estimated GAS. The cut-off value for 3D GAS is –26.75. Reducing 3D GAS by 1 increases the possibility of IUGR by 77% (Table 3).

**Table 3.** **Predictors of IUGR**.

	Univariate		Multivariable	
	OR (95% CI)	*p*-value	OR (95% CI)	*p*-value
Age (years)	1.057 (1.005–1.113)	0.032		
BMI (kg/m^2^)	0.920 (0.863–0.980)	0.010	0.751 (0.666–0.847)	<0.001
SBPav daytime (mmHg)	1.099 (1.067–1.133)	<0.001		
SBPav nighttime (mmHg)	1.101 (1.071–1.132)	<0.001		
DBPav daytime (mmHg)	1.161 (1.110–1.214)	<0.001		
DBPav nighttime (mmHg)	1.135 (1.099–1.172)	<0.001		
2D mass index (g/m^2^)	1.078 (1.051–1.106)	<0.001		
3D mass index (g/m^2^)	1.080 (1.053–1.108)	<0.001		
2D LVEDV (mL)	0.982 (0.968–0.997)	0.017		
3D LVEDV (mL)	0.983 (0.969–0.997)	0.015		
2D LV EF (%)	0.734 (0.651–0.828)	<0.001		
3D LV EF (%)	0.709 (0.633–0.795)	<0.001		
2D CO index (L/min/m^2^)	0.191 (0.100–0.368)	<0.001		
3D CO index (L/min/m^2^)	0.270 (0.153–0.479)	<0.001		
2D LV GLS	0.447 (0.340–0.588)	<0.001		
3D LV GLS	0.463 (0.367–0.584)	<0.001		
3D LV GCS	0.779 (0.651–0.933)	<0.001		
3D LV GRS	0.730 (0.662–0.806)	<0.001		
3D LV GAS	0.299 (0.214–0.417)	<0.001	0.234 (0.155–0.352)	<0.001

BMI, body mass index; SBPav, systolic blood pressure average value; DBPav, diastolic blood pressure average value; 2D, two-dimensional; 3D, three-dimensional; LVEDV, left ventricular end-diastolic volume; EF, ejection fraction; CO, cardiac output; 2D LV GLS, two-dimensional left ventricular global longitudinal strain; 3D LV GLS, three-dimensional left ventricular global longitudinal strain; 3D LV GCS, three-dimensional left ventricular global circumferential strain; 3D LV GRS, three-dimensional left ventricular global radial strain; 3D LV GAS, three-dimensional left ventricular global area strain; OR, odds ratio; CI, confidence interval; IUGR, intrauterine growth restriction.

**Fig. 4.**
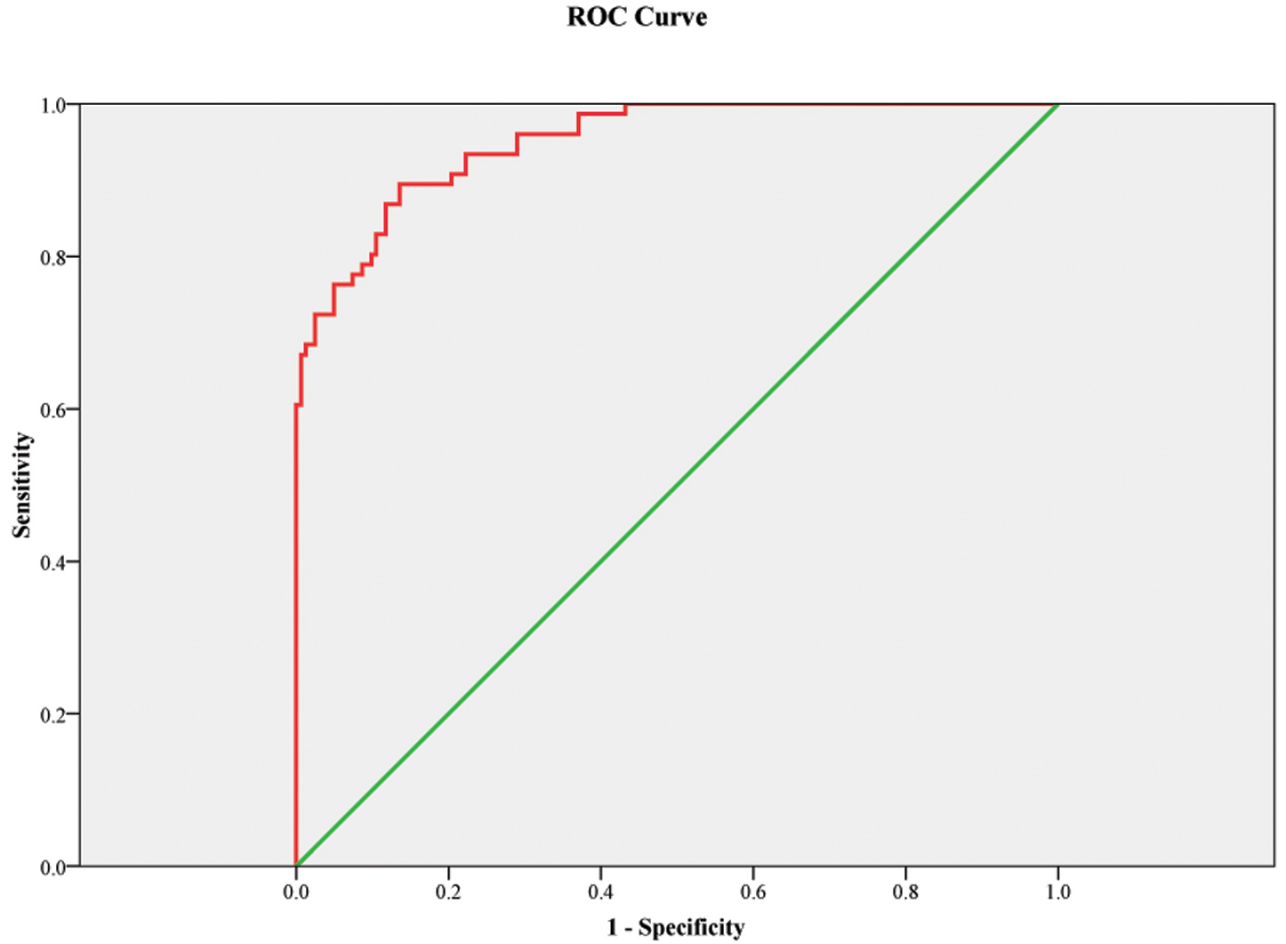
**ROC curve model for three-dimensional left ventricular global area strain as a predictor for IUGR**. Figure legend: The receiver operating characteristic curve (ROC) curve showed that this model (with BMI and 3D LV GAS, three-dimensional left ventricular global area strain) can be a good marker for the prediction of IUGR with an area under the curve 0.951; *p* < 0.001. The sensitivity is 89.5%, and the specificity is 86.4%. The green line is the reference line. BMI, body mass index; IUGR, intrauterine growth restriction.

The ROC curve showed that the 3D LV GAS can be a good predictor of IUGR. The corresponding area under the ROC curve was 0.951 (0.925–0.976) with 89.5% sensitivity and 86.4% specificity, *p* < 0.001 (Fig. 4).

#### 3.3.3 Preterm Delivery

The influence of HDP group parameters on preterm delivery was analyzed.

Multivariable analysis revealed that the three-dimensionally estimated left ventricular ejection fraction, systolic blood pressure during the night, and BMI were independent significant predictors of preterm delivery. The cut-off value for 3D LV EF is 62.05%. The 1% decrease in 3D LV EF increases the risk of preterm delivery by 22% (Table 4).

**Table 4.** **Predictors of preterm delivery**.

	Univariate		Multivariable	
	OR (95% CI)	*p* value	OR (95% CI)	*p* value
Age (years)	1.082 (1.025–1.143)	<0.001		
BMI (kg/m^2^)	0.901 (0.839–0.967)	<0.001	0.832 (0.758–0.914)	<0.001
SBPav daytime (mmHg)	1.049 (1.025–1.074)	<0.001		
SBPav nighttime (mmHg)	1.066 (1.043–1.089)	<0.001	1.055 (1.032–1.079)	<0.001
DBPav daytime (mmHg)	1.062 (1.030–1.094)	<0.001		
DBPav nighttime (mmHg)	1.071 (1.046–1.097)	<0.001		
2D mass index (g/m^2^)	1.067 (1.041–1.094)	<0.001		
3D mass index (g/m^2^)	1.067 (1.041–1.094)	<0.010		
2D LVEDV (mL)	0.974 (0.958–0.989)	<0.001		
3D LVEDV (mL)	0.972 (0.957–0.987)	<0.001		
2D LV EF (%)	0.717 (0.633–0.813)	<0.001		
3D LV EF (%)	0.732 (0.655–0.817)	<0.001	0.780 (0.687–0.885)	<0.001
2D CO index (L/min/m^2^)	0.417 (0.230–0.755)	<0.001		
3D CO index (L/min/m^2^)	0.370 (0.210–0.650)	<0.001		
2D LV GLS	0.588 (0.458–0.754)	<0.001		
3D LV GLS	0.745 (0.632–0.879)	<0.001		
3D LV GRS	0.865 (0.796–0.939)	<0.001		
3D LV GAS	0.662 (0.569–0.770)	<0.001		

BMI, body mass index; SBPav, systolic blood pressure average value; DBPav, diastolic blood pressure average value; 2D, two-dimensional; 3D, three-dimensional; LVEDV, left ventricular end-diastolic volume; EF, ejection fraction; CO, cardiac output; 2D LV GLS, two-dimensional left ventricular global longitudinal strain; 3D LV GLS, three-dimensional left ventricular global longitudinal strain; 3D LV GRS, three-dimensional left ventricular global radial strain; 3D LV GAS, three-dimensional left ventricular global area strain; OR, odds ratio; CI, confidence interval.

**Fig. 5.**
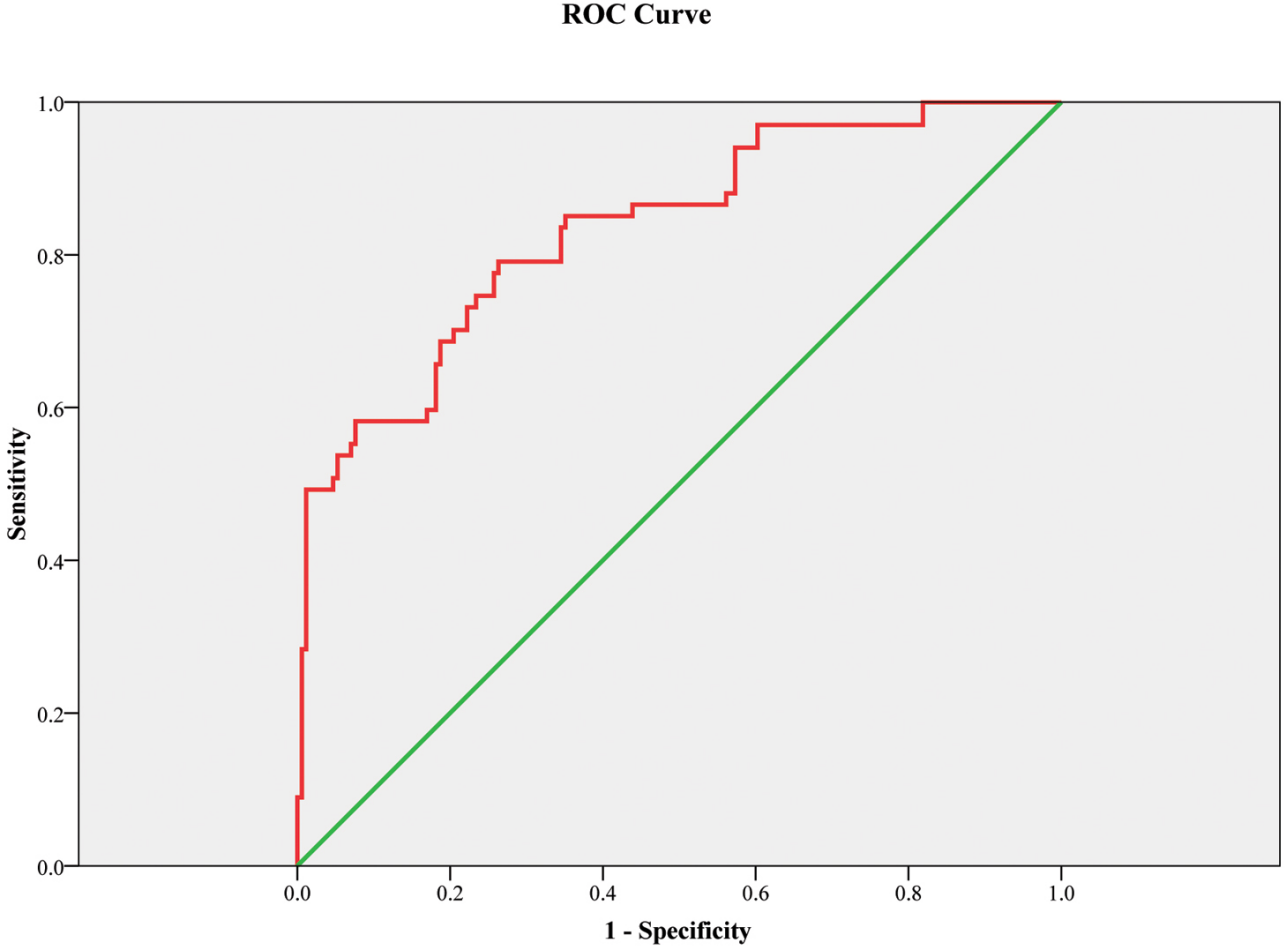
**ROC curve model for three-dimensional left ventricular ejection fraction as a predictor of preterm delivery**. Figure legend: Receiver operating characteristic curve (ROC) showed that this model (with body mass index, night-time systolic blood pressure and 3D left ventricular ejection fraction) can be a good marker for the prediction of preterm delivery. The green line is the reference line. The corresponding area under the ROC curve was 0.835 (0.776–0.893) with 79.1% sensitivity and 73.7% specificity, *p* < 0.001. 3D, three-dimensional.

The ROC curve showed that the 3D LV EF can be a good predictor of preterm delivery. The corresponding area under the ROC curve was 0.835 (0.776–0.893) with 79.1% sensitivity and 73.7% specificity, *p* < 0.001 (Fig. 5).

## 4. Discussion

Our research revealed that 3D echocardiographic assessment of the mother’s systolic function during pregnancy is a better predictor of pregnancy outcomes compared to 2DE We have shown that 3D evaluation of EF is a good predictor of preterm delivery, while 3D LV GAS is an excellent predictor of IUGR, but also correlates well with the birth weight of the newborn. To the best of our knowledge, this is the first study to assess the influence of the maternal systolic function of the LV assessed by 3DE on pregnancy outcomes.

Our findings regarding the impaired systolic and diastolic function of the left ventricle in hypertensive pregnant women, along with the increased mass of the LV compared to normotensive individuals, align with numerous previous studies [16]. Significantly lower birth weight, higher prevalence of IUGR and preterm delivery in HDP compared to normotensive pregnant women is also previously known [17]. Our research supports these findings. While changes in diastolic function and left ventricle geometry during hypertensive pregnancy have been recognized in previous decades [18, 19, 20, 21, 22, 23], data regarding systolic function remain limited and somewhat contentious [23, 24]. As mentioned in the introduction, changes in the geometry of the LV during pregnancy, necessitate a more accurate assessment of its function and morphology of the LV which can be achieved through 3DE. Cong *et al*. [6] demonstrated that both 2DE and 3DE parameters representing the function and morphology of the LV in healthy pregnant women change throughout pregnancy. They found that systolic function parameters are at their lowest during the third trimester, while the LV mass notably increases. After delivery, there is a significant improvement in systolic function parameters and LV mass decreases [6]. Our results coincide with the mentioned research for normotensive pregnant women, but also for pregnant women with HDP, where these changes were even more pronounced. Women with HDP exhibited statistically significant impairments in both 2D and 3D parameters of systolic and diastolic function of the LV, with decreases in absolute values of 2D LV GLS and 3D strain in all directions, as well as significantly higher values ​​of the LV mass index compared to normotensive pregnant women. After delivery, all the mentioned parameters showed improvement across the entire cohort, as well as distinctly in both hypertensive disorders of pregnancy groups and controls. Interestingly women who were hypertensive during pregnancy, despite being normotensive after delivery, still had statistically significant higher LV mass values compared to those who were normotensive during pregnancy.

In terms of LV systolic function, the difference in EF values was no longer significant, when assessed using both 2D and 3D methods. This change can likely be attributed to the normalization of BP during the postpartum period. Unlike LV EF, both the 2D and 3D strain values remained statistically significantly worse in women with HDP both at baseline and six weeks after delivery. This finding may imply that the assessment of the systolic function of women with HDP using the speckle tracking method may provide a more accurate indication of the subendocardial damage in the LV compared to the ejection fraction.

Company Calabuig *et al*. [25] analyzed 3D echocardiographic parameters in women with PE and showed that women with PE had lower LV diastolic function and increased LV mass index compared with controls. Notably, there was a postpartum improvement in these indices, attributed to an improvement of their risk factor profiles [25]. Pregnant women with underlying risk factors (e.g., smoking) and all other comorbidities were excluded from our research. The only exception was obesity, which was not used as an exclusion criterion. This decision was made because pregnant women were primarily screened during the third trimester, and factors such as swelling and increased volume load made BMI a less reliable exclusion metric. After delivery, we obtained the anamnestic information that some participants had actually gained more weight compared to their pre-pregnancy state, which indicates that the observed improvements in the aforementioned parameters cannot be solely attributed to the reduction of obesity as a risk factor. Although there are limited studies on the use of 3DE in pregnant women, particularly those with HDP, existing research in the non-pregnant population has demonstrated that 3D strain analysis (3D STE) offers greater accuracy and objectivity than 2D STE when assessing cardiac systolic dysfunction or dysregulation, without relying on geometric assumptions [26, 27]. Also, 3DE provides higher accuracy and reproducibility as compared to cardiac magnetic resonance both in pregnant women and in the non-pregnant population [6, 28].

The GAS, a parameter uniquely obtainable through 3D STE analysis, combines the evaluation of longitudinal and circumferential deformations. This makes it particularly sensitive to detecting anomalies without geometric assumptions, especially in the subendocardial layer, which is often one of the first areas affected in various cardiac conditions [29].

Our study showed that 3D echocardiographic assessment of the mother’s systolic function during HDP is a better predictor of pregnancy outcomes than 2D. We have shown that the strongest predictor for the occurrence of intrauterine growth retardation is a decrease in the absolute value of the GAS. Specifically, a reduction of 1 unit in GAS increases the possibility of IUGR by 77%. The cut of value of LV GAS is –26.75. Furthermore lower GAS correlates with lower birth weight. Taking into account the changes in the geometry of the LV during pregnancy, especially in hypertensive pregnant women, we may conclude that such results are expected when taking into account the volume and the geometrically independent assessment of the systolic function by the GAS.

Since similar analyzes have not been conducted in pregnant women before, we can compare them with the research demonstrating that 3D LV GLS and 3D LV GCS provide better predictive capability regarding ejection fraction and adverse events in patients with a history of myocardial infarction [30]. Additionally, LV GAS has been independently associated with an increased risk of death or heart failure following acute myocardial infarction [30]. Also, a recently published meta-analysis showed that 3D myocardial strain was reduced in all directions in asymptomatic diabetic patients compared to controls, as well as hemoglobin A1c was associated with worse 3D LV GLS and 3D LV GSC [8]. Besides that, the early change to abnormal values of 3D LV GAS was associated with a subsequent decrease in LV EF, representing a promising technique to predict chemotherapy-induced cardiomyopathy in patients with breast cancer [9].

Absolute LV GAS values ​​in our study were statistically significantly lower in hypertensive pregnant women compared to normotensive ones and remained significantly lower even after delivery, although previously hypertensive participants became normotensive. The relationship of LV GAS with BP values has been shown in a previous study [31], as well as 3D LV GLS, GCS and GRS.

Therefore we can assume that GAS, as a comprehensive parameter of myocardial systolic deformation in all three dimensions, is the most sensitive parameter for detecting occult myocardial dysfunction in HDP, as it was shown for the association with exercise time and predictive value on E/e’ for exercise capacity in participants undergoing a treadmill exercise test [32] and with common LV systolic function parameters [33].

Our study also revealed another advantage of 3D assessment of LV systolic function in pregnant women by the significant association of 3D LV EF with preterm delivery, with a cut-off value of 62.05%. The decrease of 1% in 3D LV EF increases the risk of preterm delivery by 22%. We have previously shown that 2D deterioration of LV systolic function is associated with preterm delivery [34], while a recent study showed that 3D LV EF has superior predictive ability over 2D LV EF [35]. The cut-off value of 62.05 for 3D LV EF offers valuable insights for predicting preterm delivery. However, there is a need for further clarification on its clinical application and a comparison with existing methods and established markers, such as cervical length measurement and maternal clinical factors (e.g., history of preterm birth, infections) [36]. When comparing this cut-off to other clinical methods for predicting preterm delivery, it is essential for future studies to focus on refining these predictions by considering various clinical contexts and potential combinations with other established predictors.

While traditional echocardiographic methods have been utilized to assess cardiac function in pregnancy, there is a notable scarcity of studies focusing on the specific contributions of 3DE in understanding cardiac changes in women with HDP. Our study aims to fill this knowledge gap by providing a comprehensive evaluation of 3DE parameters and their clinical relevance in this population. The application of 3DE allows for more precise and reproducible measurements of LV function without relying on geometric assumptions inherent in 2DE methods. This advancement is particularly crucial in the context of HDP, where alterations in cardiac structure and function can be subtle yet clinically significant. By utilizing 3DE , we can detect early changes that may not be apparent with conventional techniques. Our findings not only support previous research indicating cardiac dysfunction in HDP but also extend this knowledge by highlighting the utility of 3DE as a valuable tool for early detection and monitoring. The identification of specific cut-off values, such as the one we established for 3D LV EF, could be the way to integrate this into routine clinical practice.

## 5. Clinical Implications

In addition to HDP as a risk factor for cardiovascular morbidity later in life [1], a recently published study showed that preterm delivery, low birth weight of the newborn, and intrauterine growth retardation are also risk factors for future adverse maternal cardiovascular events [37]. We have shown that deterioration of the systolic function of the mother’s left ventricle during HDP obtained by 3DE assessment, is significantly related to the mentioned pregnancy outcomes. Therefore, 3D assessment of LV function during HDP, especially LV GAS, as a comprehensive parameter of myocardial systolic deformation in all three dimensions, and because of that the most sensitive parameter for detecting subclinical myocardial dysfunction in HDP, could be a good screening tool for detecting women at increased risk of developing cardiovascular complications. In that context, more frequent checks of those women are very important, as well as advising them to change their lifestyle habits.

## 6. Strengths

To the best of our knowledge, this is the first study to investigate the impact of 3DE assessment of LV systolic function in pregnant women on pregnancy outcomes.

In our view, studying 3D LV GAS is particularly significant because it combines both 3D LV GLS and 3D LV GCS. We believe this method provides a more accurate assessment of LV systolic function in pregnant women, especially those with hypertension, due to changes in LV geometry.

## 7. Limitations

It is a single-center study. Future multicenter research assessing the impact of 3D LV GAS on maternal cardiovascular events is needed to confirm our results. Also, we believe that future studies are needed to assess these parameters in earlier pregnancy (e.g., in the first trimester) in order to confirm the use of these echocardiographic measures as early markers for obstetric complications, or to potentially guide clinical decisions during the prognosis of pregnancy complications, both in terms of IUGR and preterm delivery.

## 8. Conclusions

3DE assessment of the mother’s systolic function during pregnancy is a good predictor of pregnancy outcomes. The strongest predictor of preterm delivery is 3D LV EF, while the LV GAS is the strongest predictor of the intrauterine growth retardation, but also correlates well with the birth weight.

## Availability of Data and Materials

All data reported in this paper will be shared by the lead contact upon request.
